# Human Monoclonal Antibodies against NS1 Protein Protect against Lethal West Nile Virus Infection

**DOI:** 10.1128/mBio.02440-21

**Published:** 2021-10-12

**Authors:** Alex W. Wessel, Michael P. Doyle, Taylor B. Engdahl, Jessica Rodriguez, James E. Crowe, Michael S. Diamond

**Affiliations:** a Department of Pathology & Immunology, Washington University School of Medicine, St. Louis, Missouri, USA; b Department of Medicine, Washington University School of Medicine, St. Louis, Missouri, USA; c Vanderbilt Vaccine Center, Vanderbilt University Medical Center, Nashville, Tennessee, USA; d Department of Pathology, Microbiology, and Immunology, Vanderbilt University Medical Center, Nashville, Tennessee, USA; e Department of Pediatrics, Vanderbilt University Medical Center, Nashville, Tennessee, USA; f Department of Molecular Microbiology, Washington University School of Medicine, St. Louis, Missouri, USA; g The Andrew M. and Jane M. Bursky Center for Human Immunology and Immunotherapy Programs, Washington University School of Medicine, St. Louis, Missouri, USA; Duke University Medical Center

**Keywords:** flavivirus, antibody function, epitope, viral pathogenesis

## Abstract

Envelope protein-targeted vaccines for flaviviruses are limited by concerns of antibody-dependent enhancement (ADE) of infections. Nonstructural protein 1 (NS1) provides an alternative vaccine target that avoids this risk since this protein is absent from the virion. Beyond its intracellular role in virus replication, extracellular forms of NS1 function in immune modulation and are recognized by host-derived antibodies. The rational design of NS1-based vaccines requires an extensive understanding of the antigenic sites on NS1, especially those targeted by protective antibodies. Here, we isolated human monoclonal antibodies (MAbs) from individuals previously naturally infected with WNV, mapped their epitopes using structure-guided mutagenesis, and evaluated their efficacy *in vivo* against lethal WNV challenge. The most protective epitopes clustered at three antigenic sites that are exposed on cell surface forms of NS1: (i) the wing flexible loop, (ii) the outer, electrostatic surface of the wing, and (iii) the spaghetti loop face of the β-ladder. One additional MAb mapped to the distal tip of the β-ladder and conferred a lower level of protection against WNV despite not binding to NS1 on the surface of infected cells. Our study defines the epitopes and modes of binding of protective anti-NS1 MAb antibodies following WNV infection, which may inform the development of NS1-based countermeasures against flaviviruses.

## INTRODUCTION

West Nile virus (WNV) is an enveloped, positive-sense RNA virus transmitted by *Culex* species mosquitos. Although the virus originally was endemic in parts of Africa, Europe, and Asia, it disseminated to North America in 1999, where it is now widely established, causing thousands of human and equine infections in any given year (1). Though most WNV infections remain subclinical, a subset of patients, principally the elderly and immunocompromised, develops life-threatening neurological disease. WNV can invade the central nervous system (CNS) and infect neurons in the brain and spinal cord, causing meningitis, encephalitis, and/or acute flaccid paralysis (2). Surviving patients often develop long-term sequelae that can include persistent fatigue, muscle weakness, and cognitive impairment (3, 4). No approved therapeutics or vaccines exist for WNV infection in humans.

WNV is in the *Flavivirus* genus of the *Flaviviridae* family, which includes other clinically relevant viruses, such as Japanese encephalitis (JEV), dengue (DENV), Zika (ZIKV), yellow fever (YFV), and tick-borne encephalitis (TBEV) viruses. The licensed vaccines for JEV, DENV, and TBEV target viral structural proteins and elicit neutralizing antibody responses (5, 6). The concern for possible antibody-dependent enhancement (ADE) of homologous or heterologous flavivirus infection, however, has slowed progress for envelope (E) protein-targeted vaccines and antibody therapeutics, especially for DENV and ZIKV (7, 8). Alternative strategies targeting the flavivirus nonstructural protein 1 (NS1) have been proposed to avoid ADE (9–11). NS1 is a 46- to 55-kDa glycoprotein that homodimerizes in the endoplasmic reticulum (ER) (12, 13), where it acts as a scaffold to recruit viral and host factors necessary for replication (14–17). Infected cells also express dimeric NS1 on the plasma membrane (18–20) and secrete a soluble, hexameric form of NS1 (21–25). Secreted NS1 can be detected in circulation in infected individuals (26–28) and also binds back to the surface of infected or uninfected cells through interactions with glycosaminoglycans and other receptors (29). Extracellular forms of NS1 can bind complement (30–33) and Toll-like receptors (34–36) to modulate or evade host immunity and also interact with endothelial cells to regulate permeability across blood-tissue barriers (29, 37–42).

**FIG 1:**
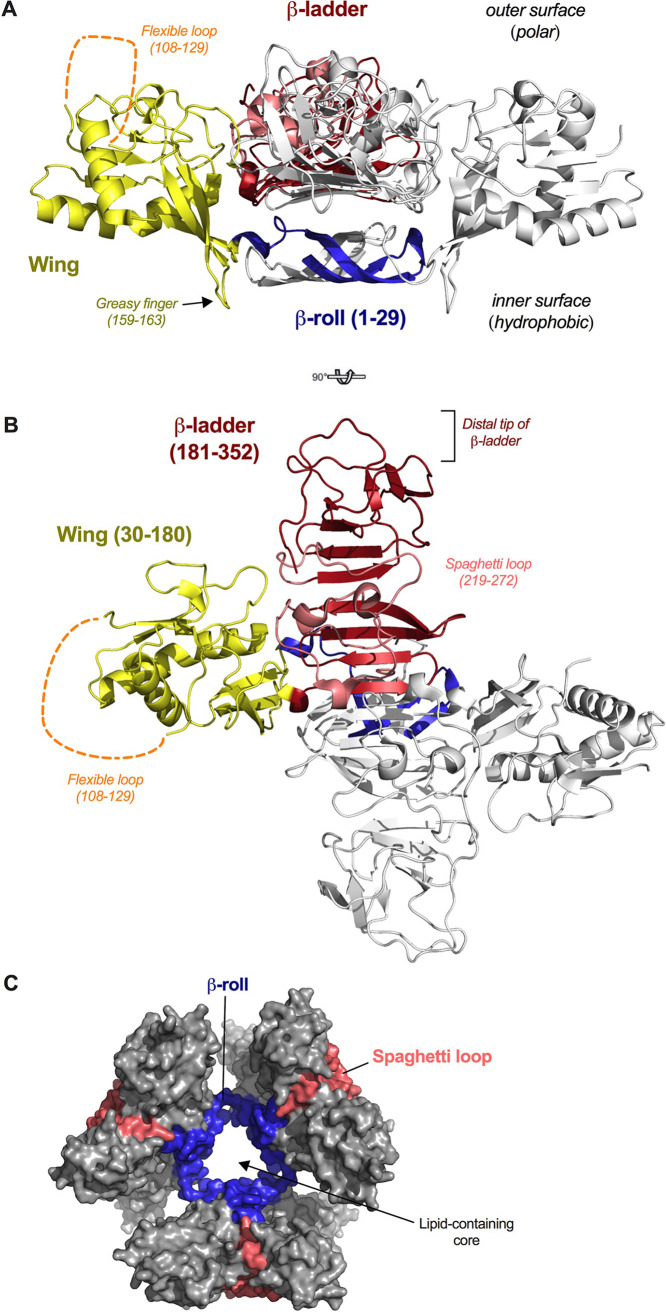
NS1 structure. (A and B) Structure of dimeric WNV NS1 (PDB 4O6D) from side (A) and top (B) views. In each structure, one monomer is gray and one is color coded by domain (blue, β-roll; yellow, wing; red, β-ladder; and salmon, spaghetti loop). The disordered flexible loop is indicated by orange dashed lines. (C) Surface representation of the DENV2 NS1 hexamer (PDB 4O6B). The hexamer core corresponds to the inner hydrophobic surfaces of each dimer protomer (β-rolls colored in blue). The outer exposed surface of the hexamer corresponds to the polar outer surfaces of the wing domains and the spaghetti loops (colored in salmon) of the β-ladder domains. This figure was prepared using PyMOL (version 2.0; Schrödinger, LLC).

The WNV NS1 dimer structure is comprised of three domains: (i) the β-roll, (ii) the wing, and (iii) the β-ladder or “β-platform” (Fig. 1A and B) (25, 43). The β-roll domains (residues 1 to 29) of two protomers intertwine and create a hydrophobic “inner surface” that mediates interactions with lipid membranes (25). The wing domains (residues 30 to 180) also contribute to this inner hydrophobic surface through two subdomains: the flexible loop (residues 108 to 129) and the “greasy finger” (residues 159 to 163) (25, 44, 45). In the hexamer (Fig. 1C), these regions create a hydrophobic inner core that packs with lipid cargo (24). The opposite “outer surface” of the wing domain contains polar residues and is more variable in sequence among flaviviruses (45). The β-ladder domain (residues 181 to 352) consists largely of β-strands that face the plasma membrane in the dimeric form of NS1. The opposite face of the β-ladder, termed the “loop face,” is covered by the spaghetti loop subdomain (residues 219 to 272), an extended loop positioned between two β-strands (25, 43).

Although extracellular NS1 is believed to contribute to pathogenesis during infection, it is also immunogenic and elicits antibody responses. NS1-specific monoclonal antibodies (MAbs) have been isolated that confer protection against challenge in animal models for YFV, DENV, ZIKV, JEV, or WNV (10, 11, 46–50). Protection can occur via Fc-dependent clearance of virus-infected cells (11, 46, 47, 51, 52) or Fc-independent mechanisms (9, 38). Extensive epitope mapping of ZIKV NS1-specific MAbs suggests that protective MAbs target a subset of surface-exposed regions in NS1, including the exposed spaghetti loop surface of the β-ladder and the electrostatic surface of the wing domain (46). For WNV, a previous study generated several protective NS1-specific murine MAbs after immunization with recombinant hexameric WNV NS1 (52). Although the epitopes for two protective MAbs have been mapped (43, 48), the breadth of epitopes in WNV NS1 that mediate protection remains largely unknown. Furthermore, no study has evaluated whether anti-NS1 human MAbs generated during natural WNV infection are like those induced in mice after immunization with recombinant proteins or whether they can confer protection. These questions are important for the development of WNV NS1-based therapeutics and vaccines. Here, we generated NS1-specific MAbs from an individual naturally infected with WNV during the 2012 outbreak in Dallas, Texas. Twelve of 13 MAbs tested conferred various degrees of protection against lethal WNV challenge in mice by limiting viral burden. Ten of the anti-WNV NS1 human MAbs that protected mice bound strongly and at high density to the surface of WNV-infected cells, whereas one nonprotective MAb (WNV-100) bound poorly to the cell surface. The epitopes of MAbs that mediated protection localized principally to the surface-exposed spaghetti loop face of the β-ladder domain, although one protective MAb (WNV-99) mapped to the wing flexible loop. Additionally, one MAb (WNV-97) conferred a lower level of protection despite not binding to cell surface-expressed NS1. As our findings suggest that human antibodies targeting specific epitopes on NS1 can protect against infection and disease, a path forward for rational design of NS1-based vaccine seems plausible.

## RESULTS

### Generation of anti-WNV NS1 human monoclonal antibodies.

**FIG 2:**
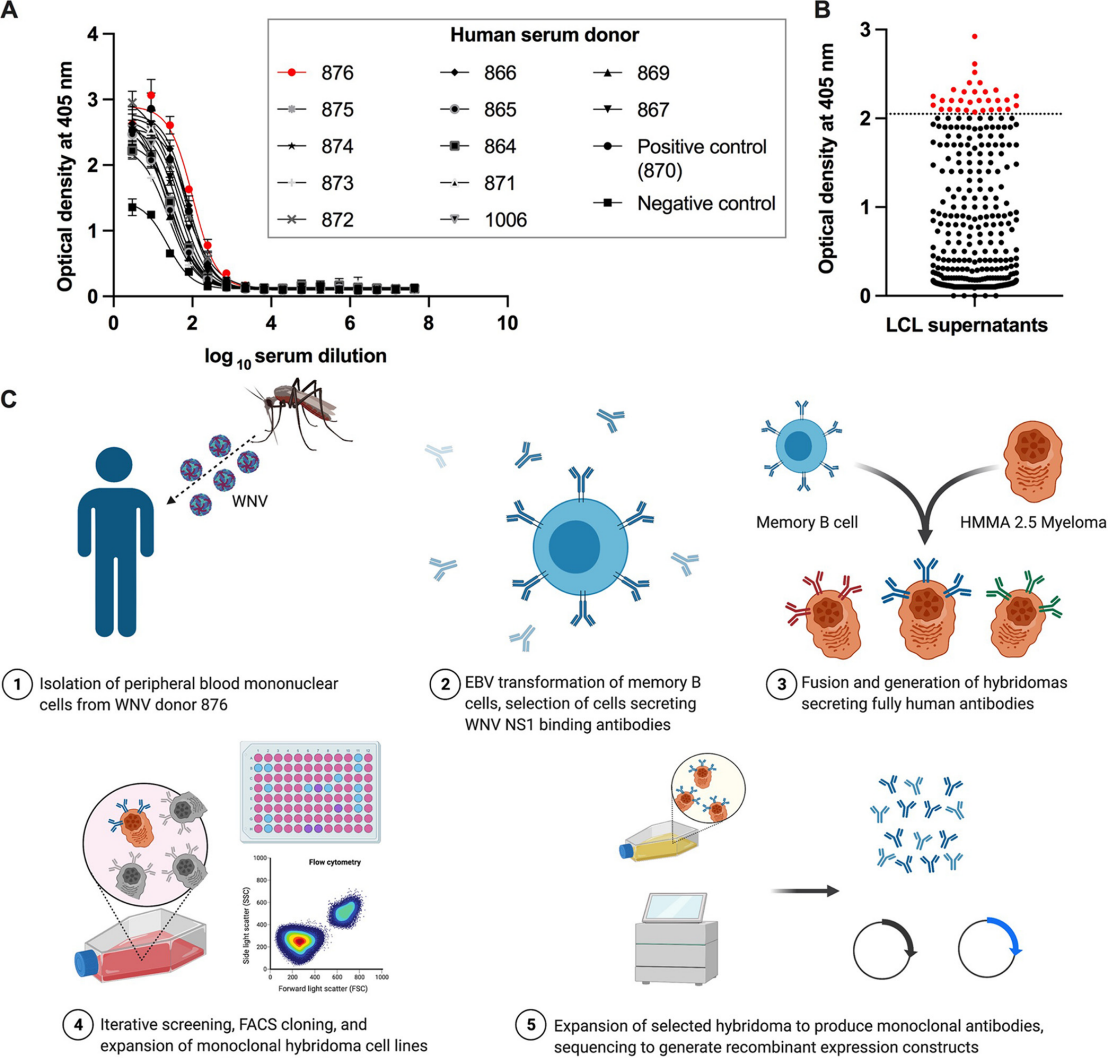
Scheme of NS1 hybridoma screen. (A) Analysis of human donor sera for binding to recombinant WNV NS1 by ELISA. A blood sample from immune individual 876 was chosen for lymphoblastoid cell line (LCL) generation and monoclonal antibody discovery. (B) Reactivity of LCL supernatants from individual 876 to WNV NS1 by ELISA. Each dot represents a different well of LCLs, and red indicates the LCLs chosen for electrofusion with HMMA 2.5 cells to generate hybridoma cell lines. (C) Full schematic of hybridoma discovery campaign used to isolate WNV NS1-specific MAbs. Panel adapted from “Monoclonal Antibodies Production” by Biorender.com (2021). Retrieved from https://app.biorender.com/biorender-templates.

We obtained matched serum and blood samples from 13 individuals with laboratory-confirmed, symptomatic WNV infection during the 2012 outbreak in Dallas, Texas (53, 54). We first screened sera for binding to recombinant WNV NS1 by ELISA and identified the individual with the highest serum anti-NS1 antibody titers (Fig. 2A). From this individual, we isolated B cells from the matched blood sample and generated lymphoblastoid cell lines (LCLs) through Epstein-Barr virus transformation. LCLs were screened by ELISA for reactivity to recombinant WNV NS1 protein, and those secreting anti-NS1 antibodies were fused with myeloma cells to generate human hybridomas (Fig. 2B and C). Thirteen hybridomas secreting NS1-specfic monoclonal antibodies (MAbs) were cloned (Table 1), and the antibody genes were sequenced (see Table S1 in the supplemental material). We analyzed the constant region sequences to determine the IgG subclass for each MAb, which revealed 11 MAbs as IgG1 and two MAbs (WNV-99 and WNV-120) as IgG3. Analysis of the variable regions confirmed that all clones expressed unique antibody molecules with different combinations of heavy and light chain genes. The variable region sequences for 12 of the MAbs were cloned into plasmids for recombinant hIgG1 expression. One MAb, WNV-106, was not cloned into an expression vector due to poor sequence recovery, although we purified this MAb from hybridoma supernatant for some studies.

**TABLE 1:** Characteristics of anti-WNV NS1 MAbs

MAb	Isotype^*a*^	Cross-reactivity^*b*^	EC_50_ (binding surface NS1)^*c*^	Competition group^*d*^	Domain localization^*e*^	Critical binding residue(s)^*f*^
WNV-120	hIgG3	J	132.3	A	Wing, distal end	K44E, N82
8NS1	mIgG1		263.6	A	Wing, distal end	T77, K80, E81, G83
WNV-99	hIgG3	J, T	294.0	B	Wing, flexible loop	G119, L123, F124, L162
16NS1	mIgG2a	J	90.3	B	Wing, flexible loop	W118, I122, L123
WNV-103	hIgG1		14.5	C	Wing, outer surface	K141
WNV-95	hIgG1		15.0	C	β-Ladder, spaghetti loop	R294
WNV-96	hIgG1		27.4	C	β-Ladder, spaghetti loop	S239, D240, R294
WNV-104	hIgG1		22.1	C	β-Ladder, spaghetti loop	S239, K261, H293, R294
WNV-117	hIgG1		33.3	C	β-Ladder, spaghetti loop	S239, D240, K261, R294
10NS1	mIgG2a		13.3	C	β-Ladder, spaghetti loop	D240, R256, R294
WNV-98	hIgG1		10.8	C	β-Ladder, loop face	R314
14NS1	mIgG2a		13.3	C	β-Ladder, loop face	G295, R314
17NS1	mIgG2a		15.9	C	β-Ladder, loop face	G295, R314
WNV-113	hIgG1		33.2	C	β-Ladder, loop face	D341, E342, K343
WNV-106	hIgG1	J, D2, Y	22.5	C		Not identified
WNV-116	hIgG1		233.0	C		Not identified
WNV-100	hIgG1	J, D2, T	115.6	D	β-Ladder, C-terminal tip	P281, L307
9NS1	mIgG1	J, D2, Z, Y, T	73.5	D	β-Ladder, C-terminal tip	T301, S304, L307
WNV-97	hIgG1	T	No binding	D	β-Ladder, C-terminal tip	I308, R339

a Human (h) IgG subclasses were determined by sequencing of the antibody gene constant regions. Murine (m) IgG subclasses were previously determined (47).

b Cross-reactivity was determined by direct ELISA using recombinant NS1 proteins: J, JEV; D2, DENV2; Z, ZIKV; Y, YFV; T, TBEV.

c EC_50_ values for binding to cell surface NS1 were determined by flow cytometry analysis of WNV-infected cells, as described in the legend to Fig. 4.

d Competition groups were determined by BLI (Fig. 3A) and ELISA (Fig. S1).

e Domain localization was determined based on competition group and the identified critical binding residues, as described in the legend to Fig. 3B.

f Critical binding residues were determined by binding to cells transfected with WT or substitution variant NS1 (Fig. 3B) and defined as substitution variants resulting in <25% binding to the MAb compared to that of WT NS1.

10.1128/mBio.02440-21.5 TABLE S1 Amino acid sequences of anti-WNV NS1 human MAbs. Download Table S1, DOCX file, 0.1 MB. Copyright © 2021 Wessel et al. 2021 Wessel et al. https://creativecommons.org/licenses/by/4.0/ This content is distributed under the terms of the Creative Commons Attribution 4.0 International license.

10.1128/mBio.02440-21.1 FIG S1 ELISA-based antibody competition. Recombinant, soluble WNV NS1 was adsorbed to plates and prebound to the indicated murine MAb first. Subsequently, the indicated human MAb was added, and the percent binding relative to binding in the absence of competition (i.e., no first MAb) was determined by ELISA. Box values are the average result of two experiments performed in duplicate. Download FIG S1, TIF file, 0.6 MB. Copyright © 2021 Wessel et al. 2021 Wessel et al. https://creativecommons.org/licenses/by/4.0/ This content is distributed under the terms of the Creative Commons Attribution 4.0 International license.

We evaluated all 13 human MAbs by ELISA for cross-reactivity with other flavivirus NS1 proteins (Table 1), including JEV, ZIKV, DENV serotype 2 (DENV2), YFV, and tick-borne encephalitis virus (TBEV). Eight MAbs were WNV-specific, whereas the other MAbs displayed various degrees of cross-reactivity. Four MAbs bound to the closely related (77% amino acid identity) NS1 of JEV (WNV-99, WNV-106, WNV-120, and WNV-100), and three of them bound to the more distantly related (43% amino acid identity) NS1 of TBEV (WNV-97, WNV-99, and WNV-100). Two MAbs cross-reacted with DENV2 NS1 (55% amino acid identity) (WNV-100 and WNV-106) and one with YFV NS1 (43% amino acid identity) (WNV-106). No MAbs cross-reacted with ZIKV NS1 protein.

### Epitope mapping.

We used biolayer interferometry (BLI) and ELISA to define competition groups between the human MAbs and several murine MAbs that were generated previously after immunizing mice with soluble WNV NS1 (Fig. 3A; Fig. S1) (47). The MAbs segregated into four competition groups. Group A consisted of only one human MAb, WNV-120, and one murine MAb, 8NS1. Group B also contained only one human (WNV-99) and one murine (16NS1) MAb. Group C consisted of nine human MAbs and four murine MAbs. Among these, WNV-103 showed unidirectional competition with other group C MAbs, and two group C MAbs (WNV-104 and WNV-113) demonstrated partial competition with group D MAbs. Group D contained two human MAbs, WNV-97 and WNV-100, and one murine MAb, 9NS1.

**FIG 3:**
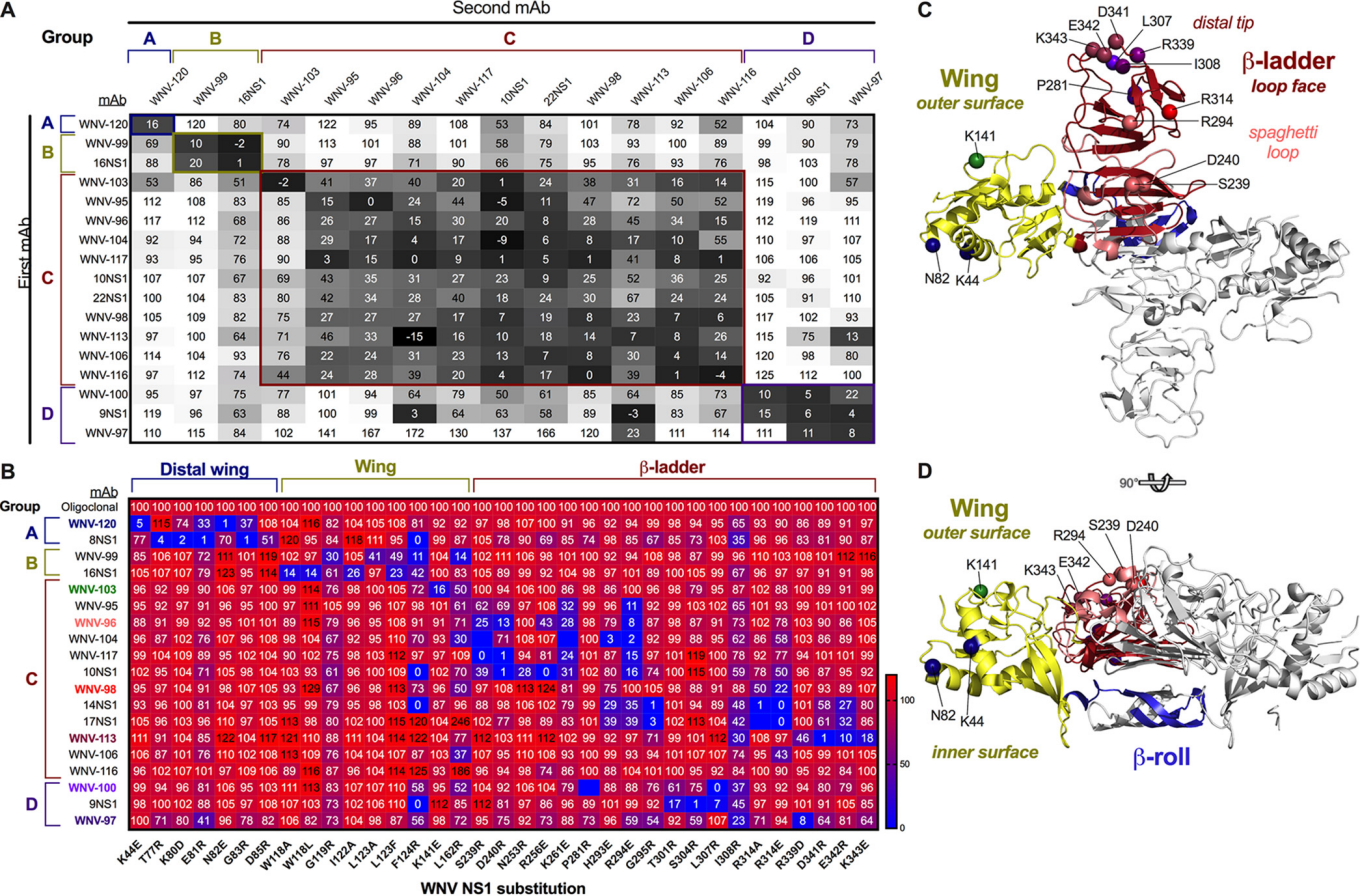
Epitope mapping. (A) Biolayer interferometry-based antibody competition for binding WNV NS1. Values represent the percent binding of the second MAb relative to its binding without a competing first MAb. Box shading indicates the degree of competition: black, strong competition (i.e., minimum residual binding); gray, intermediate competition; and white, no competition. Antibodies are grouped and labeled according to their competition group. Data are representative of one experiment. (B) Heat map of MAb binding to NS1 substitution variants relative to WT WNV NS1. 293T cells were transfected with plasmids encoding WNV NS1, and the MAb reactivity to each substitution variant relative to WT NS1 was measured by flow cytometry. Values represent the relative WT binding (mean of the results of 2 to 4 experiments), and only critical binding residues are shown (i.e., residues at which substitution resulted in less than 25% binding relative to WT NS1). (C and D) Mapping of critical binding residues onto the WNV NS1 dimer structure (PDB 4O6D) in top view (C) or side view (D). Critical residues are indicated by spheres and are color coded according to the MAb labels in panel B. In each structure, one monomer is gray and one is color coded by domain (blue, β-roll; yellow, wing; red, β-ladder; and salmon, spaghetti loop). This figure was prepared using PyMOL (version 2.0; Schrödinger, LLC).

To define the epitopes in greater detail, we mapped amino acid interaction residues of the 13 human MAbs and several of the previously published murine MAbs (47). We identified key residues for antibody binding by assessing for loss of binding to engineered charge reversal substitutions in mammalian cell-expressed WNV NS1 (strain 382-99) (Fig. 3B and Table 1). We prioritized solvent-exposed residues based on published NS1 structures (25, 43) and previously identified epitopes in the related ZIKV NS1 (46), which shares structural similarity to WNV NS1 (44, 45). In total, we generated 102 WNV NS1 mutants, each expressing a single substitution, and compared MAb binding relative to wild-type (WT) WNV NS1 using a flow cytometric assay. Residues were deemed critical for MAb binding if the substitution resulted in less than 25% binding relative to that of WT NS1 but did not affect binding by a cocktail of five anti-NS1 human MAbs from different competition groups. We found that substitution at residues K44 or N82 at the distal end of the wing domain resulted in loss of binding for the group A MAb WNV-120. The group A murine MAb 8NS1 mapped to proximal residues in this region (T77, K80, E81, and G83). Substitutions at residues G119, L123, and F124 within the wing flexible loop and at L162 within the spaghetti loop resulted in reduced binding by the group B human MAb WNV-99. The murine MAb in group B, 16NS1, mapped to adjacent residues (W118, I122, and L123) within the wing flexible loop. This latter result is consistent with previous ELISA-based peptide mapping of 16NS1 to this region (48).

The group C human MAbs mapped principally to the β-ladder domain. Notwithstanding this finding, the group C MAb WNV-103 mapped to residue K141 within the wing domain. This residue lies within the outer electrostatic surface of the wing domain and is adjacent to the spaghetti loop surface of the β-ladder (25, 44). Four group C human MAbs (WNV-95, WNV-96, WNV-104, and WNV-117) and one murine MAb (10NS1) mapped to residues within the spaghetti loop (residues 219 to 272) and loop face of the β-ladder: S239, D240, R256, K261, H293, and R294. The group C murine MAb 22NS1 also has contact residues in the spaghetti loop, as determined by a crystal structure of it bound to a C-terminal fragment of WNV NS1 (43). The group C MAbs WNV-98 and WNV-113 mapped to residues that are more C-terminal within the β-ladder, although still on the spaghetti loop face (for WNV-98, R314; and for WNV-113, D341, E342, and K343). Two murine MAbs in this competition group, 14NS1 and 17NS1, mapped to proximal residues in this region: G295 and R314. Despite substantial effort, the epitopes for group C MAbs WNV-106 and WNV-116 were not identified using the charge reversal substitutions. In contrast, both human MAbs and the one murine MAb in group D mapped to the distal tip of the β-ladder domain (for WNV-97, I308 and R339; for WNV-100, P281 and L307; and for 9NS1, T301, S304, and L307).

Residues identified as key for binding were mapped onto the WNV NS1 crystal structure (Fig. 3C and D). Notably, the four competition groups localized to distinct regions on the structure. Group A MAbs bound at the distal end of the wing domain, adjacent to the binding site for the group B MAbs within the wing flexible loop. Antibodies in group C localized primarily to the spaghetti loop surface of the β-ladder, except for WNV-103, which mapped to the adjacent outer surface of the wing. Both regions are predicted to be exposed on the cell surface form of NS1 (25, 43). Group D MAbs localized to the tip of the β-ladder domain, at residues on or approximating the membrane-facing β-strand surface. As such, these residues may be less accessible on the cell surface form of NS1.

### NS1 binding properties.

**FIG 4:**
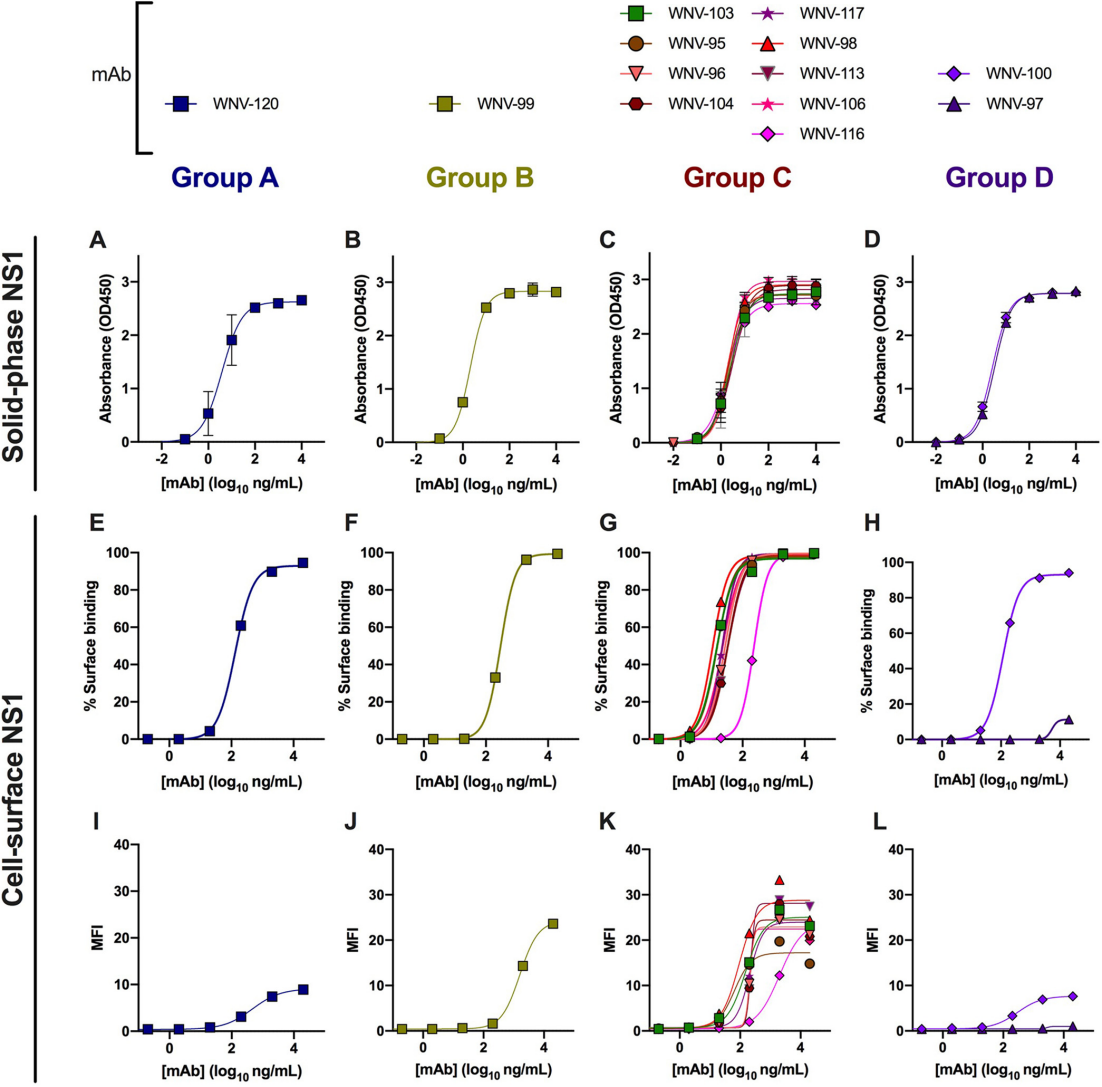
Binding properties of anti-WNV NS1 human MAbs. (A to D) Binding to recombinant, soluble WNV NS1 was determined by ELISA. Absorbance values are the average of duplicates from two experiments. (E to L) Binding to cell surface-associated NS1 on live, WNV-infected Vero cells was assessed by flow cytometry. The percentage of cells staining (E to H) or the mean fluorescence intensity (MFI) (I to L) is shown as the average result of two experiments. Error bars represent the mean ± standard deviation (SD). EC_50_ values for binding to cell surface-associated NS1 are reported in Table 1. Data are separated by antibody competition group: panels A, E, and I, group A; panels B, F, and J, group B; panels C, G, and K, group C; and panels D, H, and L, group D.

Given that NS1 is expressed as a dimer on the cell surface and as a soluble hexamer in the extracellular space and in circulation during infection, we evaluated the binding of the human and murine MAbs by using three methods. First, we assessed binding to solid-phase recombinant WNV NS1 protein by ELISA. All human and murine MAbs bound avidly (half-maximal effective concentration [EC_50_], ∼1 to 5 ng/ml) (Fig. 4A to D, human MAbs; Fig. S2A, murine MAbs). Next, we measured binding to NS1 expressed on the surface of intact, WNV-infected Vero cells using flow cytometry (Fig. 4E to H and Table 1). The MAbs in groups A and B bound infected cells relatively similarly (EC_50_ for WNV-120, 132 ng/ml, and for WNV-99, 294 ng/ml) (Fig. 4E and F). Within group C, WNV-95, WNV-98, and WNV-103 bound most avidly (EC_50_ for WNV-95, 15 ng/ml; WNV-98, 11 ng/ml; and WNV-103, 15 ng/ml), whereas most other group C MAbs bound slightly less efficiently (EC_50_, ∼20 to 30 ng/ml) (Fig. 4G). One exception was WNV-116, which bound approximately 10- to 20-fold less avidly (EC_50_, 233 ng/ml) than the other group C MAbs. The group D MAb WNV-100 (EC_50_, 116 ng/ml) bound with a strength similar to that of WNV-116 and the MAbs in groups A and B (Fig. 4H). The group D MAb WNV-97, which mapped to a residue on the membrane-facing surface of the β-ladder, failed to bind to the surface of infected cells. We also compared the densities of surface binding by the human MAbs by using the mean fluorescence intensity (MFI) (Fig. 4I to L). On average, the group C MAbs bound to infected cells with the highest site density (Fig. 4K), except for WNV-99 in group B (Fig. 4J). The MAbs in groups A and D bound at a lower density to infected cells (Fig. 4I and L). Finally, we performed biolayer interferometry to assess binding of the anti-WNV NS1 human MAbs to the soluble, hexameric form of WNV NS1. All of the MAbs engaged the hexamer, although WNV-97 bound less well than the other anti-NS1 MAbs (Fig. S3).

10.1128/mBio.02440-21.2 FIG S2 Binding properties of anti-WNV NS1 murine MAbs. (A) Binding to recombinant, soluble WNV NS1 was determined by ELISA. Absorbance values are the average of duplicates and representative of two experiments. (B and C) Binding to cell surface-associated NS1 on live, WNV-infected Vero cells was assessed by flow cytometry. The percentage of cells staining (B) or the MFI (C) is shown as the average result of two experiments. Error bars represent the mean ± SD. EC_50_ values for binding to cell surface-associated NS1 are reported in Table 1. Antibodies are grouped and labeled according to their competition group. Download FIG S2, TIF file, 0.3 MB. Copyright © 2021 Wessel et al. 2021 Wessel et al. https://creativecommons.org/licenses/by/4.0/ This content is distributed under the terms of the Creative Commons Attribution 4.0 International license.

10.1128/mBio.02440-21.3 FIG S3 Binding of anti-WNV NS1 human MAbs to hexameric NS1. The anti-WNV NS1 human MAbs were biotinylated and bound to streptavidin-coated pins for 300 s using biolayer interferometry. After a baseline measurement, association of the MAbs to recombinant, hexameric NS1 (250 nM to 3.9 nM) was determined for 300 s, followed by a dissociation step for 300 to 600 s. A biotinylated isotype control MAb (hu-CHK-152; black line) was used as a negative control. Data are from one experiment. Download FIG S3, TIF file, 0.9 MB. Copyright © 2021 Wessel et al. 2021 Wessel et al. https://creativecommons.org/licenses/by/4.0/ This content is distributed under the terms of the Creative Commons Attribution 4.0 International license.

For comparison, we also measured the surface binding for published anti-WNV NS1 murine MAbs (Fig. S2B and C) (47). The avidity of surface binding varied by competition group, as seen with the human MAbs. The group C murine MAbs 10NS1, 14NS1, 17NS1, and 22NS1 all bound avidly and to a similar extent as the human MAbs in group C. Most MAbs in groups A, B, and D bound the cell surface less avidly and at lower density than those in group C. However, the group B MAb 16NS1 bound with a similar density as the group C MAbs, although with less avidity.

### Protection by anti-WNV NS1 human MAbs.

**FIG 5:**
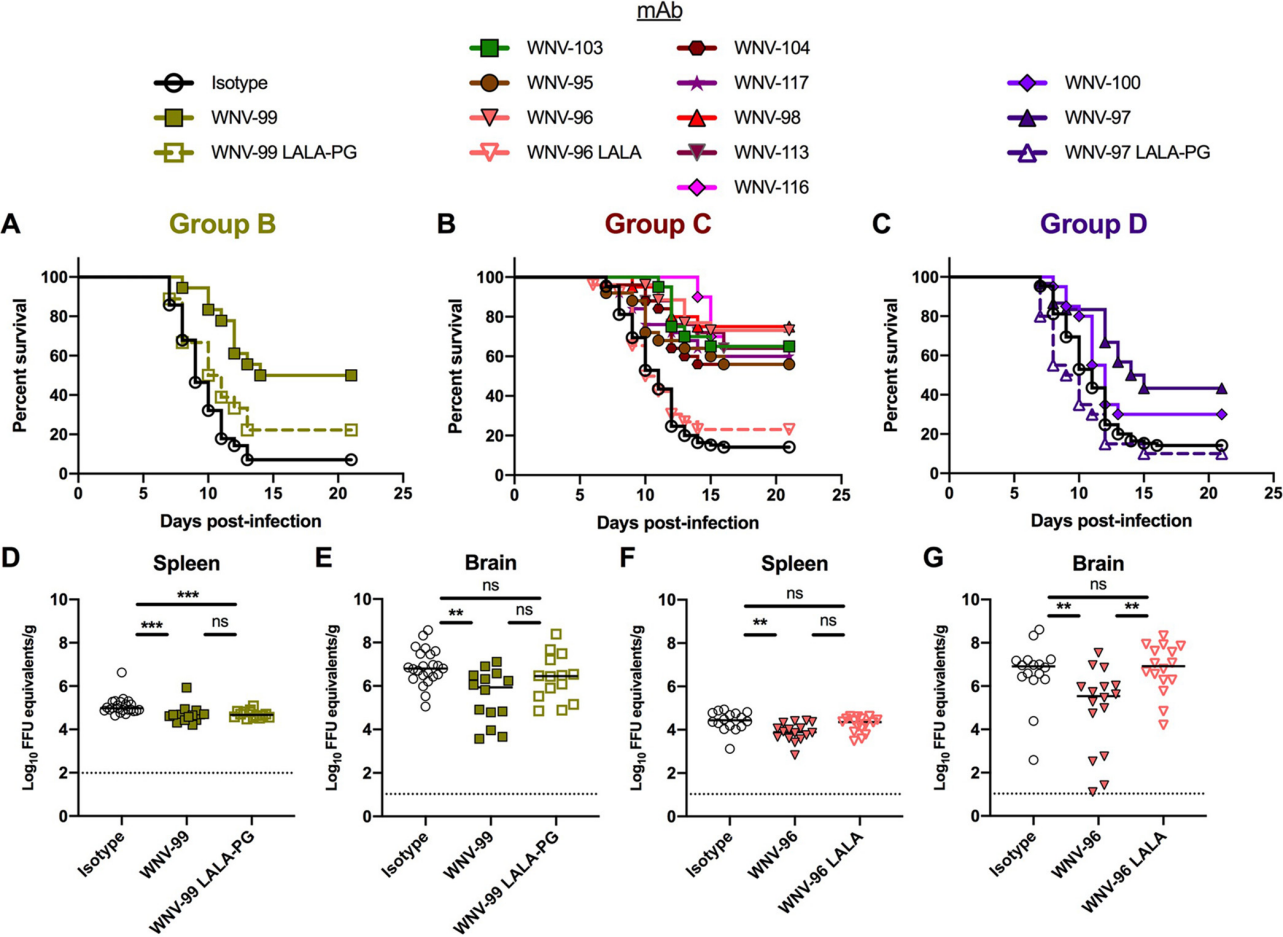
Protection against WNV challenge. (A to C) Survival analysis. Four- to 5-week-old male C57BL/6J mice were inoculated subcutaneously with 10^2^ FFU of WNV and concurrently administered 200 μg of anti-WNV NS1 human MAb or an isotype control MAb. (A) Survival data for the group B MAb WNV-99 was analyzed using the log rank Mantel-Cox test (isotype, *n* = 28, 4 experiments; WNV-99, *P* < 0.0001, *n* = 18, 2 experiments; WNV-99 LALA-PG, *P* ≥ 0.99, *n* = 18, 2 experiments). (B and C) Survival for the MAbs in group C (B) and group D (C) were analyzed together using the log rank Mantel-Cox test with a Bonferroni correction (isotype, *n* = 85, 11 experiments; WNV-95, p 0.0024, *n* = 25, 3 experiments; WNV-96, *P* < 0.0001, *n* = 26, 4 experiments; WNV-96 LALA, *P* ≥ 0.99, *n* = 26, 4 experiments; WNV-97, *P* = 0.0036, *n* = 30, 3 experiments; WNV-97 LALA-PG, *P* ≥ 0.99, *n* = 20, 2 experiments; WNV-98, *P* < 0.0001, *n* = 20, 2 experiments; WNV-100, *P* ≥ 0.99, *n* = 20, 2 experiments; WNV-103, *P* < 0.0001, *n* = 20, 2 experiments; WNV-104, *P* < 0.0001, *n* = 25, 3 experiments; WNV-113, *P* < 0.0001, *n* = 25, 3 experiments; WNV-116, *P* < 0.0001, *n* = 20, 2 experiments; WNV-117, *P* < 0.0001, *n* = 25, 3 experiments). (D to G) Viral burden analysis. Mice were inoculated with 10^2^ FFU of WNV and concurrently administered 200 μg of isotype control MAb (hu-CHK-152) or anti-NS1 MAb: WNV-99 and WNV-99 LALA-PG (D and E) or WNV-96 and WNV-96 LALA (F and G). At 7 days postinfection (dpi), viral RNA levels were determined by qRT-PCR in the spleen (D and F) and brain (E and G). (D to G) Viral burden data were analyzed using Kruskal-Wallis one-way ANOVA with Dunn’s posttest: panels D and E, isotype, *n* = 23; WNV-99, *n* = 14; WNV-99 LALA, *n* = 14; isotype versus WNV-99, *P* = 0.0003 (D) and *P* = 0.0024 (E); isotype versus WNV-99 LALA-PG, *P* = 0.0007 (D) and *P* = 0.3491 (E); panels F and G, all MAbs, *n* = 16; isotype versus WNV-96, *P* = 0.0021 (F), and *P* = 0.01 (G); isotype versus WNV-96 LALA, *P* = 0.7437 (F) and *P* ≥ 0.99 (G); WNV-96 versus WNV-96 LALA, *P* = 0.0762 (F) and *P* = 0.0047 (G).

We next assessed the ability of anti-WNV NS1 human MAbs to protect against virus challenge in mice (55). Four- to 5-week-old C57BL/6J male mice were inoculated subcutaneously with 10^2^ focus-forming units (FFU) of WNV (New York strain 382-99) and concurrently administered a single 200-μg (10 mg/kg) dose of antibody. We did not evaluate WNV-120 in group A due to its poor stability in solution or the group C MAb WNV-106 because it was not cloned successfully into an expression vector. Whereas only ∼10% to 15% of mice administered an isotype control antibody (hu-CHK-152) (56) survived infection over 3 weeks, the NS1-specific human MAbs conferred various degrees of protection (Fig. 5). The group B MAb WNV-99 conferred ∼50% protection (Fig. 5A). The group C MAbs conferred the greatest protection, resulting in 56% to 75% survival rates (Fig. 5B). The group D MAb WNV-97 conferred ∼43% protection, whereas WNV-100 did not protect against mortality (Fig. 5C). The protection was Fc dependent for at least three MAbs (WNV-96, WNV-97, and WNV-99), as demonstrated by a loss of activity with IgG1-LALA or IgG1-LALA-PG Fc variants that abrogate binding to most Fcγ receptors and complement (57). We confirmed loss of binding by WNV-96 LALA, WNV-97 LALA-PG, and WNV-99 LALA-PG to human Fcγ receptor I by ELISA (Fig. S4).

10.1128/mBio.02440-21.4 FIG S4 Fcγ receptor I ELISA. Binding of WNV-96 and WNV-96 LALA at the indicated concentrations to recombinant human Fcγ receptor I by ELISA. Values are the average of duplicates. Download FIG S4, TIF file, 0.4 MB. Copyright © 2021 Wessel et al. 2021 Wessel et al. https://creativecommons.org/licenses/by/4.0/ This content is distributed under the terms of the Creative Commons Attribution 4.0 International license.

For two protective MAbs in groups B (WNV-99) and C (WNV-96), we evaluated the effects on viral burden. Four- to 5-week-old C57BL/6J mice were inoculated subcutaneously with 10^2^ FFU of WNV (strain 382-99) and concurrently administered 200 μg (∼10 mg/kg) of MAb (Fig. 5D to G). At 7 days post-infection (dpi), we assessed the viral RNA levels in the spleen and brain. WNV-99 and WNV-96 resulted in small reductions in viral RNA in the spleen and conferred larger reductions in the brain than the isotype control MAb. The protective effect in the brain for both MAbs was lost using IgG1-LALA or IgG1-LALA-PG variants, suggesting Fc effector function-dependent mechanisms of protection (Fig. 5E and G).

## DISCUSSION

In this study, we show that human anti-WNV NS1 MAbs that bind with the greatest strength and site density to cell surface-expressed NS1 confer the highest levels of protection against WNV infection. Antibody-mediated protection in mice and avid cell surface binding were associated with epitopes on the loop face of the β-ladder domain (group C), which is exposed on cell surface forms of NS1. However, one protective group C human MAb (WNV-103) mapped to the outer electrostatic surface of the wing domain, another exposed epitope on cell surface NS1. We also mapped several previously generated, protective murine MAbs (10NS1, 14NS1, and 17NS1) within group C to the loop face of the β-ladder (47). Our data are consistent with a recent study of anti-ZIKV NS1 MAbs, which found that protective MAbs mapped to exposed epitopes on cell surface forms of NS1, including the spaghetti loop face of the β-ladder and outer surface of the wing domain (46). Protection by anti-ZIKV NS1 MAbs was Fc dependent, which is consistent with our data for at least one group C MAb (WNV-96). Anti-NS1 MAbs binding to cell surface NS1 may facilitate Fc-mediated clearance of WNV-infected cells, such as through phagocytosis by macrophages (52) or classical complement-mediated cytolysis (19, 50). Avid binding with high site density to infected cells may be required to cross-link bound antibodies and promote Fc effector functions (58–61).

One human (WNV-99) and one murine (16NS1) MAb in group B (47, 48), which mapped to the wing flexible loop, also conferred protection. In comparison to the group C human MAbs, however, WNV-99 yielded less protection and bound less avidly to NS1 on the cell surface. Two previously generated anti-ZIKV NS1 MAbs (Z12 and Z13), which also mapped to the wing flexible loop, failed to protect against ZIKV infection and pathogenesis (46). These MAbs, however, bound with even lower density to cell surface forms of NS1 than 16NS1 or WNV-99. Notably, the wing flexible loop is comparatively less disordered in the crystal structure for ZIKV NS1 than WNV NS1 (25, 44, 45). The flexible loop structure in ZIKV NS1 forms a hydrophobic protrusion or “spike” that is thought to facilitate association with the plasma membrane (38, 44, 45). Potentially, this region is more flexible in WNV NS1 to allow for greater MAb binding to NS1 on the cell surface. Alternatively, the angle of binding may differ between the anti-ZIKV and anti-WNV NS1 MAbs such that the plasma membrane sterically inhibits binding of some anti-ZIKV NS1 MAbs. It is also possible that substitution at the identified residues for these two MAbs might disrupt binding through allosteric effects rather than direct effects on contact residues. High-resolution structural studies using X-ray crystallography or cryo-electron microscopy of WNV-99 or 16NS1 Fab fragments bound to WNV NS1 may be needed to definitively characterize this epitope.

The human MAbs in groups A (WNV-120) and D (WNV-97 and WNV-100) bound less efficiently to NS1 on the cell surface than group C MAbs. Whereas WNV-97 failed to bind the surface of infected cells entirely, WNV-120 and WNV-100 bound at lower site density than group C MAbs. Based on the NS1 dimer structure, the critical binding residues for the group A MAbs span the inner and outer surfaces at the distal tip of the wing domain (25). Potentially, the binding orientation of the MAbs may be angled toward the inner surface, leading to steric hindrance by the cell plasma membrane. Consistent with the relatively poor accessibility of their epitopes on the surface of infected cells, WNV-100 or WNV-97 conferred no or low levels of protection *in vivo*, respectively. The residual protection conferred by WNV-97, despite lacking binding to the infected cell surface, could be explained by blocking of pathogenic functions of soluble forms of NS1, including effects on endothelial permeability (37, 39, 40). Indeed, residues at the tip of the β-ladder (near the epitope for WNV-97) are critical for inducing endothelial hyperpermeability by DENV NS1 (38, 42). Furthermore, two MAbs against DENV NS1 mapping to this region blocked NS1-induced endothelial hyperpermeability and protected mice against DENV infection (9, 38). Some group C MAbs such as 14NS1, WNV-98, or WNV-113 might be expected to act through similar mechanisms, given that their epitopes are proximal to the tip of the β-ladder domain of WNV NS1.

Several of the anti-WNV NS1 MAbs demonstrated various degrees of cross-reactivity with other flavivirus NS1 proteins. The group B MAbs 16NS1 and WNV-99 both bound JEV NS1 and mapped to the wing flexible loop, which contains several highly conserved residues (e.g., W115, K116, W118, and G119). This region could be conserved due to its importance in association of NS1 with cell membranes (38, 44, 45). All three MAbs in group D (9NS1, WNV-97, and WNV-100) bound at least one heterologous flavivirus NS1 protein. This result is consistent with the epitope location of these MAbs at the conserved regions in the tip of the β-ladder domain (25, 45). At least three other previously generated, protective NS1-specific MAbs (749-A4 [46], 2B7 [38], and 1G5.3 [9]) also map to the distal tip of the β-ladder and cross-react with multiple flavivirus NS1 proteins. Two of these MAbs (2B7 and 1G5.3) block DENV NS1-mediated endothelial hyperpermeability and protect against DENV pathogenesis without requiring Fc effector functions (9, 38). Thus, the tip of the β-ladder might be a candidate site for immune focusing efforts to generate protective, cross-reactive MAbs against multiple flaviviruses (62–64). Nonetheless, several reports suggest that epitopes at the tip of the β-ladder of DENV NS1 (residues ∼305 to 330) can induce autoantibodies that react with human endothelial cells, platelets, and coagulation factors (65–71). In some mouse models, these MAbs induce thrombocytopenia, coagulopathy, and plasma leakage (70, 71). Thus, further studies are warranted to determine the safety of MAbs binding to this conserved, C-terminal region of NS1. Alternatively, targeting of other epitopes in NS1 (e.g., the outer surface of wing domain or spaghetti loop) may provide protection without the risk of adverse consequences. Vaccines could be designed to focus the immune response on specific epitope locations, either through design of NS1 immunogens lacking the conserved C-terminal region or by masking these epitopes by adding additional N-linked glycans in a site-specific manner (72–75).

Our studies examined antibody-based protection against the New York 1999 (NY99) genotype of WNV. However, several other WNV genotypes, such as NA/WN02 and SW/WN03, have been identified in North America (76, 77). Compared to NY99, the other genotypes have only three amino acids in NS1 that vary consistently (A70S, S99P, and L206F) (76–78). Since none of our MAbs map to these residues, they also should confer protection against strains from these genotypes. One study sequenced more recent WNV isolates from the 2012 epidemic in Dallas, Texas, and identified only one amino acid change in NS1 (I236V) that appeared in more than one isolate (79). Given that I236V is a conservative substitution and that our MAbs do not map to this residue, our anti-NS1 MAbs likely bind these isolates as well. Nonetheless, newer strains of WNV with changes in NS1 sequences that affect binding by some of our protective MAbs could evolve.

In summary, we isolated a panel of NS1-specific MAbs from a human subject naturally infected with WNV and defined three main epitopes associated with protection: (i) the wing flexible loop, (ii) the outer, electrostatic surface of the wing, and (iii) the loop face of the β-ladder (including the spaghetti loop and adjacent C-terminal loop face). Most protective epitopes were associated with avid binding to NS1 on the surface of infected cells, with the caveat that a subset of MAbs may confer protection without binding to the cell surface. One limitation of this study is that we analyzed only 13 NS1-specific MAbs from one individual. A larger panel of MAbs from multiple human donors might enable a more extensive characterization of functionally important epitopes in WNV NS1 and provide greater resolution to structure-function analyses.

## MATERIALS AND METHODS

### Ethics statement.

**(i) Animal procedures.** Animal procedures were performed in accordance with the recommendations in the *Guide for the Care and Use of Laboratory Animals* of the National Institutes of Health. Protocols were approved by the Institutional Animal Care and Use Committee at Washington University School of Medicine (assurance no. A3381-01). To minimize suffering during procedures, animals were anesthetized with ketamine hydrochloride and xylazine.

**(ii) Human subjects.** In 2014, blood samples were collected from adults with a history of symptomatic, laboratory-confirmed WNV infection during the 2012 outbreak in Dallas, Texas (54). The study was approved by the Institutional Review Board of Vanderbilt University Medical Center, and samples were obtained by the Vanderbilt Clinical Trials Center following written informed consent from each subject.

### Cell culture.

BHK-21 (American Type Culture Collection [ATCC], CCL-10), C6/36 (ATCC, CRL-1660), HEK-293T (ATCC, ACS-4500), and Vero cells (ATCC, CCL-81) were propagated in Dulbecco’s modified Eagle medium (DMEM) supplemented with 10% fetal bovine serum (FBS), 1 mM sodium pyruvate, and 10 mM HEPES, pH 7.3. Raji-DCSIGNR cells (80) were propagated in RPMI 1640 medium supplemented with 7% FBS, GlutaMAX (Invitrogen), and 100 U/ml penicillin/streptomycin (Invitrogen). DCSIGNR expression on Raji-DCSIGNR cells was confirmed using mouse anti-human DC-SIGNR/CD299 (10 μg/ml; R&D Systems, clone MAB162, 120604). Cells were maintained under the following conditions: C6/36 cells, 28°C and 5% CO_2_; Raji-DCSIGNR cells, 37°C and 7% CO_2_; all other cells, 37°C and 5% CO_2_.

### Production of infectious WNV.

We generated recombinant, infectious WNV using a two-plasmid (pWN-AB and pWN-CG) infectious clone for WNV-NY99, strain 382-99 (GenBank accession no. AF196835) (81, 82). The pWN-AB and pWN-CG plasmids were purified from SURE 2 competent cells (Agilent), digested with NgoMIV and XbaI, and then treated with alkaline phosphatase. Following phenol-chloroform extraction and precipitation with ethanol, the DNA fragments were joined with T4 DNA ligase at 16°C overnight. The assembled DNA was subsequently linearized with XbaI (5 h at 37°C) and treated with proteinase K (30 min at 50°C). After purification by phenol-chloroform extraction and ethanol precipitation, the resulting DNA was used as a template for *in vitro* transcription using the AmpliScribe T7 high-yield transcription kit (Lucigen). RNA product was electroporated into BHK-21 cells using a GenePulser Xcell electroporator (Bio-Rad) at 850 V, 25 mF, and infinite Ω. The P0 virus stocks were recovered within 4 days and propagated in C6/36 cells to generate P1 virus stocks. Titers of virus stocks were determined by focus-forming assay (FFA) using Vero cells (83).

### Generation of anti-WNV NS1 human antibodies.

The WNV-immune human donors were described previously (54). Peripheral blood mononuclear cells (PBMCs) were isolated by gradient centrifugation of heparinized blood layered on Ficoll Histopaque. Subsequently, B cells from donor 876 were transformed by incubation in medium containing Epstein-Barr virus (obtained from the supernatant of B95.8 cells, ATCC), 2.5 μg/ml CpG (phosphorothioate-modified oligodeoxynucleotide ZOEZOEZZZZZOEEZOEZZZT; Life Technologies), 10 μM Chk2 inhibitor (Sigma), and 10 μg/ml cyclosporine (Sigma). After incubation in 384-well plates for 7 days, the B cells were expanded into four 96-well plates containing CpG, Chk2 inhibitor, and irradiated heterologous human PBMCs (to serve as feeder layers for lymphoblastoid cell clusters). After 3 days, supernatants (5 μl per well) were screened by ELISA for binding to recombinant soluble WNV NS1 protein (Native Antigen).

For wells with anti-NS1 antibody, cells were subjected to electrofusion with HMMA 2.5 myeloma cells (84). The fused cells were then cultured for 14 to 18 days in selective medium containing 100 μM hypoxanthine, 0.4 μM aminopterin, 16 μM thymidine (HAT medium supplement; Sigma HO262) and 7 μg/ml ouabain (Sigma O3125). Hybridomas were screened by ELISA for anti-WNV NS1 antibody, and cells from positive wells were cloned by sorting live, single cells into 384-well plates using a FACSAria III cell sorter (Becton, Dickinson). Cloned cells were cultured for about 14 days and screened by ELISA again for NS1-specific antibody. For purification of antibody from hybridoma clones, cells were cultured in serum-free medium (hybridoma SFM; Life Technologies) for 21 days. Antibody in the supernatant was captured by affinity chromatography on HiTrap MabSelect SuRe columns (Life Technologies) according to the manufacturer’s protocol. Antibodies subsequently were eluted from the affinity columns and concentrated using Amicon centrifugal filters (Millipore). Antibody heavy and light chain variable genes were amplified by reverse transcription (RT)-PCR from hybridoma cell RNA and subjected to Sanger sequencing. Complementary DNAs representing the coding regions of antibody variable genes were synthesized and cloned into expression vectors for recombinant human IgG1, IgG1-LALA, or IgG1-LALA-PG. Recombinant antibodies were expressed in 293F cells.

### Recombinant antibody purification.

To generate recombinant antibodies, mRNA was isolated from hybridoma cell lines, and heavy and light chain gene sequences were amplified using 5′ RACE (rapid amplification of cDNA ends). A Pacific Biosciences instrument was then used to sequence amplicon libraries. To express recombinant antibodies, heavy and light chain genes were cloned into pML or Twist plasmid expression vectors. Constructs were transfected into ExpiCHO cells using ExpiFectamine CHO reagent according to the vendor’s instructions (ThermoFisher Scientific). After 7 to 8 days, cell supernatants were harvested and clarified through 0.45-μm filters. Antibody was then purified by affinity chromatography (ÄKTA pure; Cytiva) using HiTrap MabSelect SuRe (IgG1) or protein G (IgG3) columns (Cytiva). Eluted antibody was quenched immediately in Tris buffer and buffer-exchanged into PBS by centrifugation or dialysis.

### Epitope mapping.

Epitope mapping was performed using charge reversal mutagenesis at targeted residues in WNV NS1. A mammalian expression vector, pFM-A1.2, was constructed to encode full-length WNV NS1 (strain 382-99), preceded by the cluster of differentiation 33 (CD33) signal sequence and followed in frame with a 6× histidine affinity tag. The pFM-A1.2 expression vector was subjected to site-directed mutagenesis (GenScript or GENEWIZ) to generate a library of 102 total mutants. Hydrophobic and negatively charged residues were replaced with arginine, and positively charged residues were replaced with aspartic acid or glutamic acid. In a few cases, residues were replaced with alanine. HEK293T cells were transfected with each substitution variant plasmid using Lipofectamine 3000 (ThermoFisher) and incubated at 37°C for 1 day to allow for NS1 expression. Cells were fixed in 4% paraformaldehyde (PFA) in PBS for 10 min at room temperature and washed in PBS containing 2 mM EDTA and 0.2% bovine serum albumin (BSA) (FACS buffer). Subsequently, cells were incubated for 1 h at 4°C with individual MAbs or a cocktail of 5 anti-WNV NS1 MAbs (WNV-96, WNV-98, WNV-100, WNV-113, and WNV-116) as a control for NS1 protein expression. After washing, cells were incubated for 30 min at 4°C in Alexa Fluor 647 conjugated to goat anti-human or goat anti-mouse IgG (1:2,000 dilution; ThermoFisher Scientific). Flow cytometry was performed on a MACSQuant analyzer (Miltenyi Biotec). Based on previously published criteria (46, 85), critical binding residues for each MAb were defined as substitution variants with <25% reactivity relative to the WT protein. Only variants with >70% reactivity (compared to the WT) to the oligoclonal antibody pool were considered for epitope mapping. Critical binding residues were mapped onto the NS1 dimer structure (PDB 406D) using PyMOL software (version 2.3.4; Schrödinger).

### Biolayer interferometry-based antibody competition.

Competition-binding studies were performed using a FortéBio HTX biolayer interferometry instrument. HIS1K sensor tips (FortéBio lot no. 1907172) were soaked in wells containing recombinant WNV NS1 at 5 μg/ml, diluted in kinetics buffer (Pall lot no. 6090032) for 180 s. After a brief baseline step, a first antibody then was associated with coated sensor tips at 100 μg/ml for 600 s to achieve complete saturation. After another baseline step, the tips were soaked in a second antibody at 100 μg/ml for 180 s. Binning data were analyzed using FortéBio Data Analysis HT software (version 11.1.2.48). A buffer-only control was used to normalize all antibody binding. The competition groups were defined using a Pearson correlation analysis (embedded in Data Analysis HT software).

### Antibody competition-binding ELISA.

MaxiSorp 96-well microtiter plates (Nunc) were coated overnight at 4°C with 20 ng of recombinant WNV NS1 protein (Native Antigen) in 50 μl of sodium bicarbonate buffer, pH 9.3. Subsequently, plates were washed four times with PBS and blocked with ELISA buffer (PBS containing 2% BSA and 0.05% Tween 20) for 1 h at 37°C. Plates then were incubated with anti-WNV NS1 murine MAbs at 10 μg/ml for 1 h at room temperature. Without washing, anti-WNV NS1 human MAbs were added to the plates at preoptimized concentrations and incubated for 10 min at room temperature. The plates then were washed four times in PBS containing 0.05% Tween 20 and incubated with goat anti-human IgG conjugated to horseradish peroxidase (1:2,000 dilution; Jackson ImmunoResearch 109-035-088) for 30 min at room temperature. After washing, plates were developed using 3,3',5,5'-tetramethylbenzidine substrate (Agilent) for 5 to 10 min. The reaction was stopped using 2 N H_2_SO_4_, and absorbance (450 nm) was read using a TriStar microplate reader (Berthold Technologies).

### NS1 ELISA.

Fifty nanograms of WNV, JEV, DENV2, ZIKV, YFV, or TBEV NS1 proteins (all from Native Antigen) were immobilized on MaxiSorp microtiter plates (Nunc) overnight at 4°C in 50 μl of sodium bicarbonate buffer, pH 9.3. The plates were washed four times with PBS and blocked with ELISA buffer by incubation for 1 h at 37°C. Subsequently, the plates were incubated with MAb (anti-WNV NS1 or isotype control) diluted in ELISA buffer for 1 h at room temperature. For cross-reactivity ELISAs, the plates were incubated with MAb at 1 μg/ml; for avidity studies, the plates were incubated with serial dilutions of MAb as indicated in the figures. After washing four times in PBS containing 0.05% Tween 20, the plates were incubated with biotinylated goat anti-human or goat anti-mouse IgG (H+L; 1:2,000 dilution; Jackson ImmunoResearch) for 1 h. The plates were washed again and incubated with streptavidin-conjugated horseradish peroxidase (1:625 dilution; Vector Laboratories) for 15 min. After a final wash series, the plates were developed, and absorbance was read as described above. For avidity studies, the EC_50_ of binding to solid-phase NS1 was calculated using a 4-parameter logistic curve.

### Binding to cell surface NS1.

Vero cells were inoculated with WNV at a multiplicity of infection (MOI) of 5. After 24 h, the cells were washed in PBS and detached by incubation in PBS containing 10 mM EDTA for 15 min at 37°C. The cells were washed in chilled FACS buffer and pelleted by centrifugation at 300 × *g* for 3 min at 4°C. Subsequently, the cells were resuspended in serial dilutions (20 μg/ml to 2 pg/ml) of MAb (anti-NS1 or isotype control) for 1 h at 4°C. After washing in FACS buffer, cells were stained with fixable viability dye eFluor 506 (1:1,000 dilution; eBioscience) and Alexa Fluor 647 conjugated to goat anti-human or anti-mouse IgG (1:2,000 dilution; ThermoFisher). Cells were washed again before fixing in 4% PFA in PBS for 10 min and then processed on a MACSQuant analyzer (Miltenyi Biotec). After gating on live cells, the percent reactivity to cell surface-expressed NS1 was determined for each dilution of MAb. The EC_50_ of binding to cell surface NS1 was calculated using a 4-parameter logistic curve.

### Biolayer interferometry assay for hexameric NS1.

The anti-WNV NS1 MAbs and an isotype control MAb (hu-CHK-152) were biotinylated using a commercial kit (ThermoFisher A39256) and purified in PBS using desalting columns (ThermoFisher 89890). Binding of the MAbs to recombinant, hexameric WNV NS1 (Native Antigen) was evaluated using a FortéBio HTX biolayer interferometry instrument. Streptavidin sensor tips (FortéBio) were soaked in wells containing the biotinylated MAbs at 5 μg/ml in kinetics buffer (HBS-EP buffer [0.01 M HEPES pH 7.4, 150 mM NaCl, 3 mM EDTA, and 0.005% vol/vol surfactant P20] containing 3% BSA) for 300 s. After a brief baseline step, serial dilutions of recombinant WNV NS1 (250 nM to 3.9 nM) were incubated with the MAb-coated sensor tips for 300 s. Subsequently, dissociation was allowed to proceed for 300 to 600 s. Data were analyzed using FortéBio Data Analysis HT software (version 11.1.2.48).

### Fcγ receptor I ELISA.

MaxiSorp 96-well plates were coated overnight at 4°C with 35 ng of recombinant human Fcγ receptor I (CD64) protein (R&D Systems) in 50 μl of sodium bicarbonate buffer, pH 9.3. Plates then were washed and blocked as described above. Serial dilutions of the IgG1-WT and -LALA variants of WNV-96 were added to the plates and incubated for 2 h at room temperature. Subsequently, the plates were washed and incubated with goat anti-human IgG conjugated to horseradish peroxidase (1:2,000 dilution; Jackson ImmunoResearch 109-035-088) for 45 min. The plates then were washed and developed as described above for the NS1 ELISAs.

### Mouse experiments.

Animal studies were performed using 4- to 5-week-old C57BL/6J male mice (Jackson Laboratory 000664). Mice were inoculated by subcutaneous injection in the footpad with 10^2^ FFU of WNV (New York strain 382-99) in a 50-μl volume. Concurrently, mice were administered MAb (anti-WNV NS1 or an isotype control, hCHK-152) (56) by intraperitoneal injection. Mice were euthanized after 21 days or on day 7 for assessment of virus titers in specific organs. Mice were perfused with 20 ml of PBS prior to organ harvest.

### Viral burden analysis.

Mouse organs were weighed and homogenized by zirconia bead dissociation using a MagNA Lyser (Roche) in a volume of DMEM containing 2% FBS. RNA was isolated from the tissue homogenates using either the RNeasy 96 kit (Qiagen) or the MagMAX-96 viral RNA isolation kit (Applied Biosystems). WNV RNA levels were determined by TaqMan one-step quantitative reverse transcriptase PCR (qRT-PCR) using the following primer and probe sequences: forward primer, 5′-GGGTCAGCACGTTTGTCATTG-3′; reverse primer, 5′-TCAGCGATCTCTCCACCAAAG-3′ probe, 5′-TGCCCGACCATGGGAGAAGCTC-3′ (83). An RNA standard curve was generated using a defined viral stock to calculate FFU equivalents from RNA levels.

### Data analysis.

The statistical tests for each data set are indicated in the respective legends and were performed using Prism version 8 software (GraphPad). The EC_50_ of MAb binding to solid-phase (ELISA) and cell surface-expressed (flow cytometry) NS1 was determined by nonlinear regression using a 4-parameter logistic curve. Survival curves were analyzed by the log rank Mantel-Cox test with a Bonferroni correction. To compare viral titers in specific organs between MAb groups, Mann-Whitney tests or one-way analyses of variance (ANOVAs) with Dunn’s posttest were performed. Statistical significance was defined as a *P *of <0.05. FlowJo software version 10 (Becton, Dickinson and Company) was used to analyze all flow cytometry data sets.

### Data availability.

All primary raw data will be made available upon request. Antibodies will be made available under a Material Transfer Agreement with Vanderbilt University.
