# Supplementary material for: Human Monoclonal Antibodies against NS1 Protein Protect against Lethal West Nile Virus Infection
Source: mBio. 2021 Oct 12;12(5):e02440-21. doi: 10.1128/mBio.02440-21 (PMC8510529; doi:10.1128/mBio.02440-21)
Supplement: TABLE S1 [file mbio.02440-21-st001.docx]

| **Table S1. Amino acid sequences of anti-WNV NS1 human mAbs** | | |
| --- | --- | --- |
| **MAb** | **Heavy Chain** | **Light Chain** |
| WNV-95 | QVQLQESGPGLVKPSQTLSLTCTVSGGSINSGAFYWSWIRQHPGKGLEWIGFIYYSGSTYYNPSLRSRVTISVDTSKNQFSLKLISVTAADTAVYYCARDRPDDPGPYFDSWGRGTLVTVSS | QSALTQPASVSGSPGQSITISCTGTSNDVGRYNSVSWYQQHPGNAPKLMIYDVSNRPSGVSNRFSGSKSGNTASLTISGLQAEDEADYYCSSYTSSSSVVFGGGTKLTVL |
| WNV-96 | QVQLVQSGAEVKKPGASVKVSCRPSGYIFTNYYIEWVRQAPGQGLEWMGIINPSGGKTTYAQKFQGRVTMTSDTSTSTVYMELSSLRSDDTAVYYCARDGNGSDYWGQGTRVTVSS | DVQMTQSPSSLSASVGDRVTITCRASQNINNYLNWYQQKPGRAPKLLIYATSNLESGVPSRFSGSRSGADFTLTISSLQPEDFASYYCQQSYNAPRTFGHGTKVEIK |
| WNV-97 | QVQLVESGGGLVQPGGSLRVSCAASGFTFSTYEMNWVRQAPGKGLEWVSYISNSGTIIYYADSVKGRFTISRDNAKNSLYLQMNSLRAEDTAIYYCGRVLRDRDVVAGPPARVWDYYYYGMDVWGQGTTVTVSS | DIVMTQSPLSLPVTPGEPASISCRSSQSLLHSSGYNCLDWYLQKPGQSPQLLIYLRSNRASGVPDRFSGSGSGTDFTLKISRVEAEDVGVYYCMQTLQTPWTFGQGTKVEIK |
| WNV-98 | QVQLVESGGGLVKPGGSLRLSCAASGFPFTNYYMSWIRQAPGKGLEWISYITSRGDTAYYADSVKGRFTVARDNSKNSLYLQMNSLRAEDTAMYFCARGGDDYADSWGQGILVTVSS | EIVLTQSPATLPLSPGERATLSCRASQSINTNLAWYQQKPGQAPRLLIYETSHRASAIPARFSGSGSGTDFTLKISSLEPEDLAIYYCQQYRNWPQTFGQGTRLEIK |
| WNV-99 | QVRLVESGGGVVQPGRSLRLSCAASGFTLSSYGMHWVRQAPGKGLEWVAIISYDGSDKYYADSVKGRFTISRDNSKNTLYLQMNSLRPEDTAMYYCAKPDRSSSPFAHWGQGTLVTVSS | SYELTQPPSVSVSPGQTARITCSGDALPKQYAFWYQQKPGQTPVVVISKDSERPSGIPERFSGSSSGTTVTLTISGVQAEDEADYYCQSADSSGIWVFGGGTKLTVL |
| WNV-100 | QLQLQESGSGLVKPSQTLSLTCTVSGDSIGSGGFSWTWIRQPPGKGLEWIGNIYHSGGTYYNPSLKSRVTISIDNSTHFSLKLSSVTAADSAVYYCARDRGMVIKRRPWSYGLDVWGQGTTVSVSS | QSVLTQPPSASGTPGQRVTISCSGGSSNIGSNSVSWYQHLPRTAPKLLIYSNNQRSSGVPDRFSGSKSGTSASLAISGLQSDDEADYYCAAWDDSLHVLFGGGTKLTVL |
| WNV-103 | EVQLVESGGGLLQPGGSLRLSCAASGFSFSRYWMHWVRQAPGKGLIWVSRINTDGSTTIYADSVKGRFTVSRDNAKNTLFLQMSRLRPEDTAVYYCARVIASPGISYGMDVWGHGTTVTVSS | SHELTQPPSVSVSPGQTATITCSGDALPKQYAYWYQQKSGQAPVLVIYKDTKRPSGIPERFSGSSSGTVVTLTISGVQAEDEADYYCQSADISSTYVVFAGGTKLTVL |
| WNV-104 | QVQLVESGGGVVQPGTSLRLSCAASGFTFRSYGMHWVRQAPGKGLEWVALIWYDGSNKYYADSVKGRFTISRDNSRSTLYLQMNSLRAEDTAVYYCARDLGEYDSLVGPLYTARLGYWGQGTLVTVSS | DIQMTQSPSSLSASVGDRVTMTCQASQDISNYLNWYQQKPGKAPNLLIYDASNLETGVPSRFSGSGSGTDFTFTISSLQPEDIATYYCQQYDNLPITFGQGTRLEIK |
| WNV-113 | QITLKESGPTLVKPTQTLTVTCTFSGFSLNTNGVGVGWIRQPPGKALEWLALIYWDDVKRYRPSLESRLTITKDTSKNQVVLTMTDMDPVDTGTYYCAHRFHSRGWYTFDYWGQGTRVTVSS | SYVLTQPPSVSVAPGQTARITCGGNNIGGKNVHWYQQKPGQAPVLVVYDDNDRPSGIPERFSGSNSGNTATLTISRVEAGDEADFYCQVWDSNSEHVVFGGGTKLTVL |
| WNV-116 | QMQLVESGGGVVQPGRSLRLSCVASGFNFKTYVMNWVRQAPGKGLEWVAVILSDGDNKYYADSVKGRFTISRDNSKNTLFLQMDSLRADDTAVYYCTRVPHCSTSSCYKEYYNYYMDVWGKGTTVSVSS | EIVLTQSPATLSLSPGDRATLSCRASQSLSTSLAWYQQKPGQAPRLVIYDASNRAADIPARFSGSGSGTDFTLTIRSLEPEDFAVYYCQQRSNWPPRYTFGQGTKLEIK |
| WNV-117 | EVQLLESGGGLVQPGGSLRLSCAASGFTFSNYAMSWVRQAPGKGLEWVSSISDRGDYTYYADSVKGRFTISRDKSRNTLYLQIKSLRAEDTAVYYCAKCWGRGSYSGIPDYWGQGTLVTVSS | DIQMTQSPSSLSASVGDRVTISCQASHDITNFLNWYQQKPGKAPKLLIYDASNLEAGVPSRFSGSGSGTDFTFTISSLQPEDIATYYCQQYDNLFITFGQGTRLEIK |
| WNV-120 | KVQLEESGGGWVKPGRSLRLSCGASGFRFDDYAMHWVRQVPGKGLEWVSGISWDSDGIGYADSVKGRFTISRDNAKNSLFLQMNSLRAEDTALYYCVKDKGWLIQGRFDSWGQGIRVTVSS | DIVVTQSPDSLAVSLGERVTINCKTSQSVLYTFNNQNYLAWYQQKSGQPPKLLIYWASTRESGVPDRFSGSGSGTDFTLTISRLQAEDVAVYYCQQFYISPPGTFGQGTKVEIK |
