# Supplementary figures and images for: Human Monoclonal Antibodies against NS1 Protein Protect against Lethal West Nile Virus Infection
Source: mBio. 2021 Oct 12;12(5):e02440-21. doi: 10.1128/mBio.02440-21 (PMC8510529; doi:10.1128/mBio.02440-21)

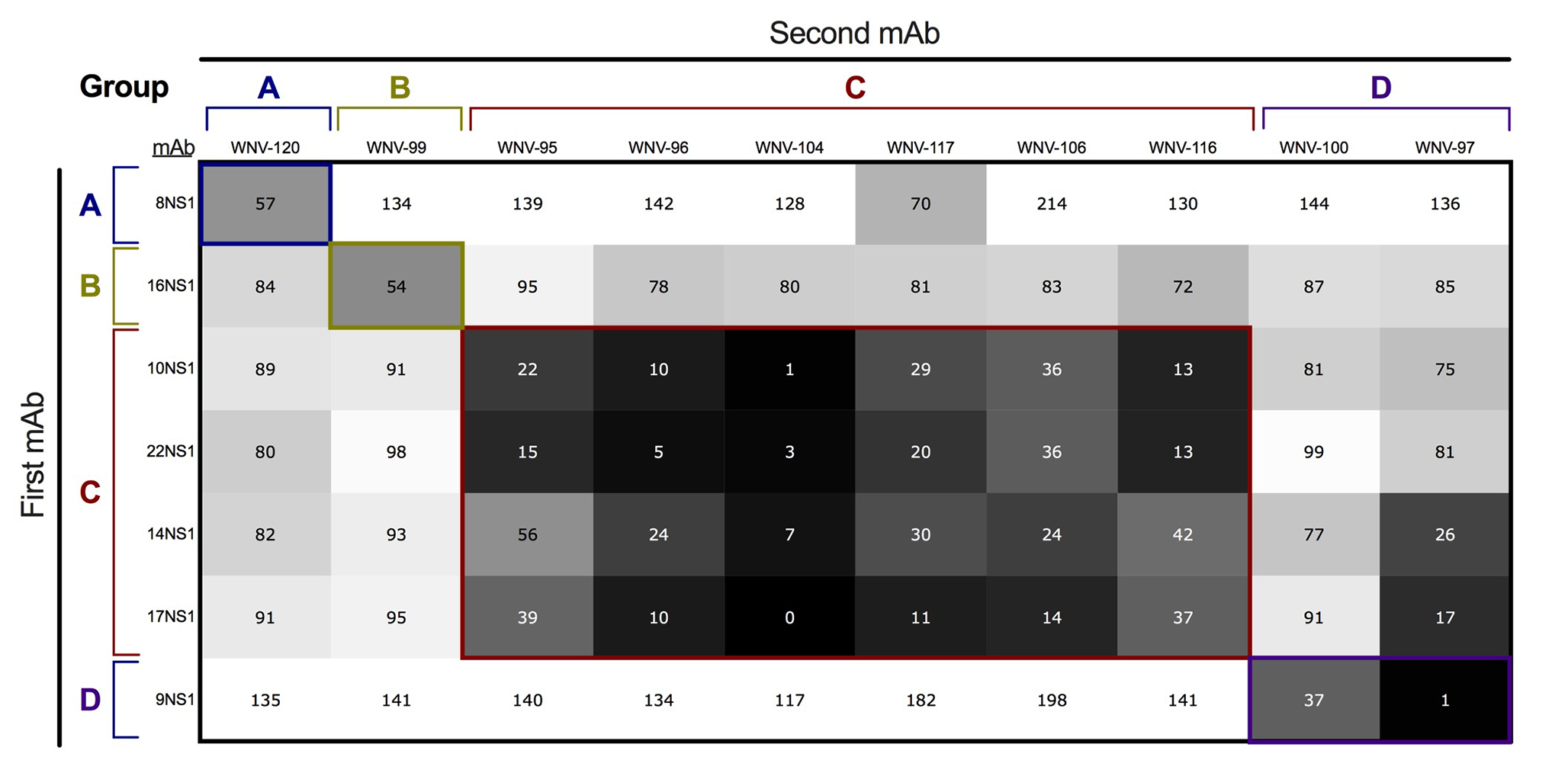

Supplement: FIG S1 [file mbio.02440-21-sf001.tif]

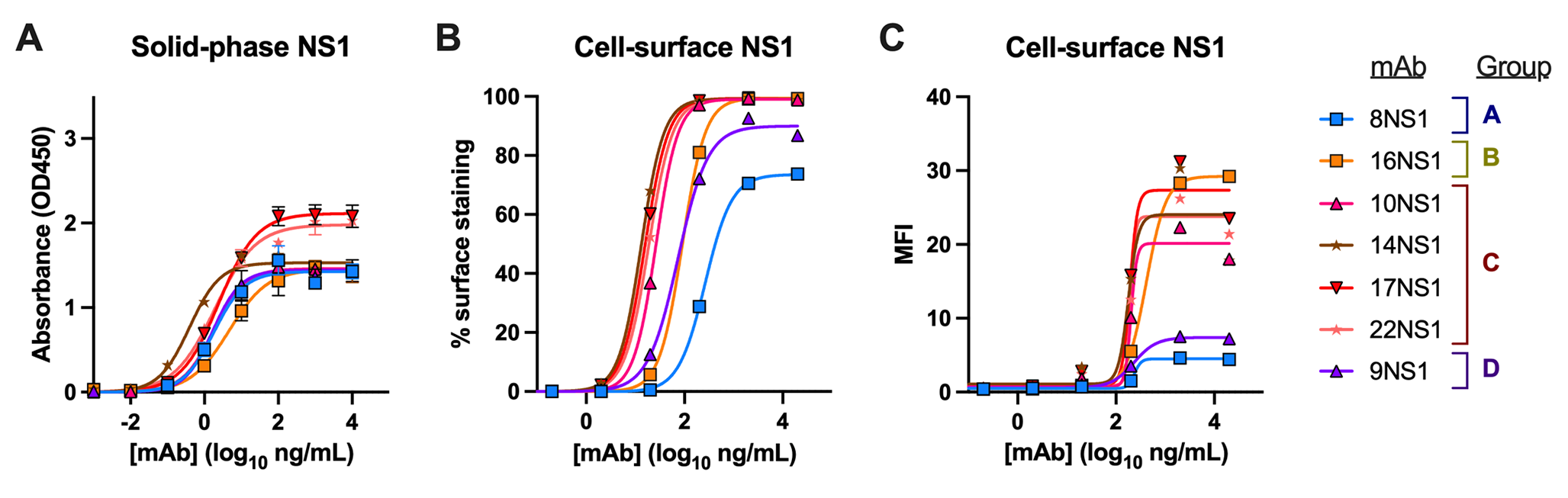

Supplement: FIG S2 [file mbio.02440-21-sf002.tif]

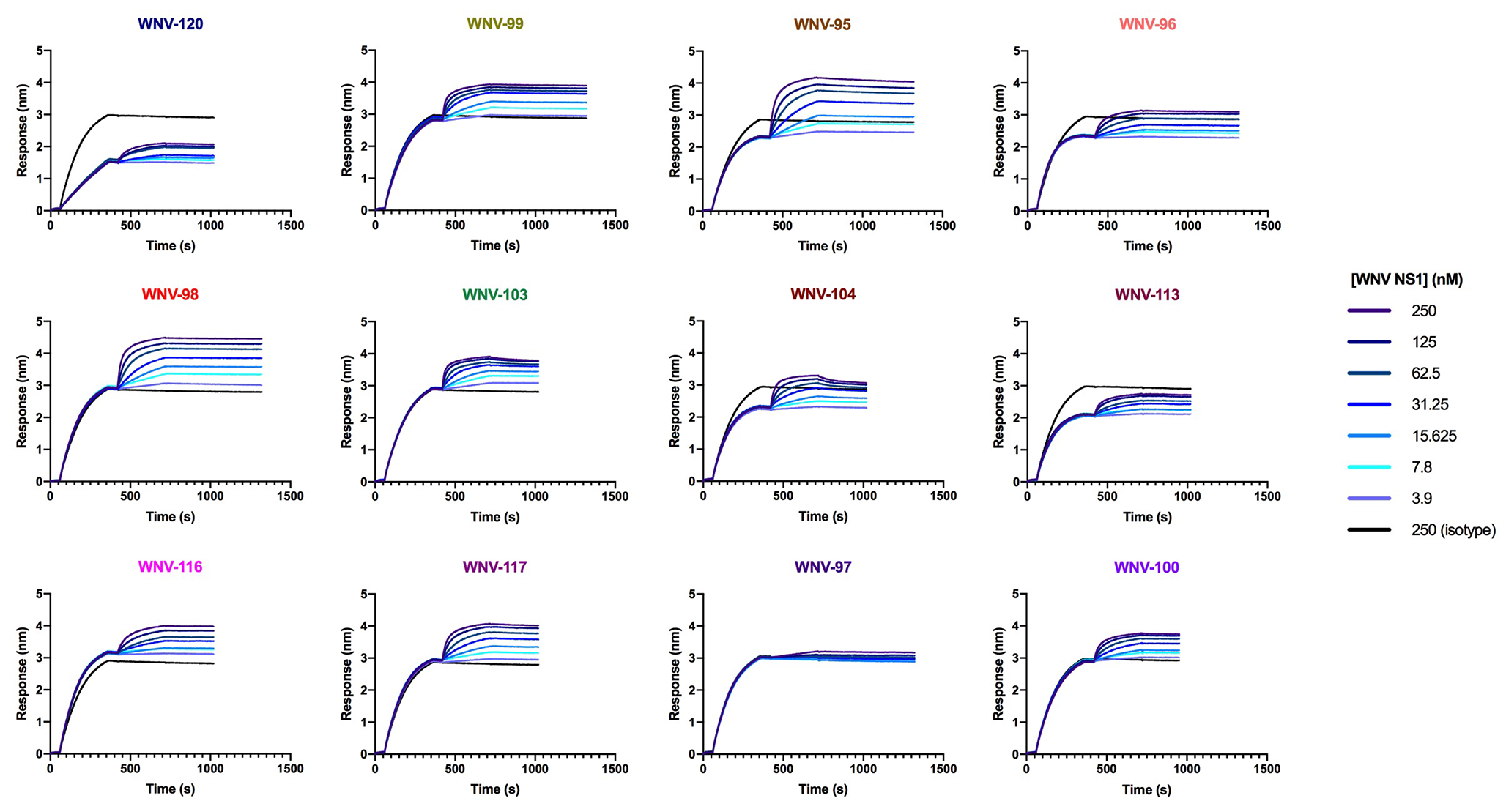

Supplement: FIG S3 [file mbio.02440-21-sf003.tif]

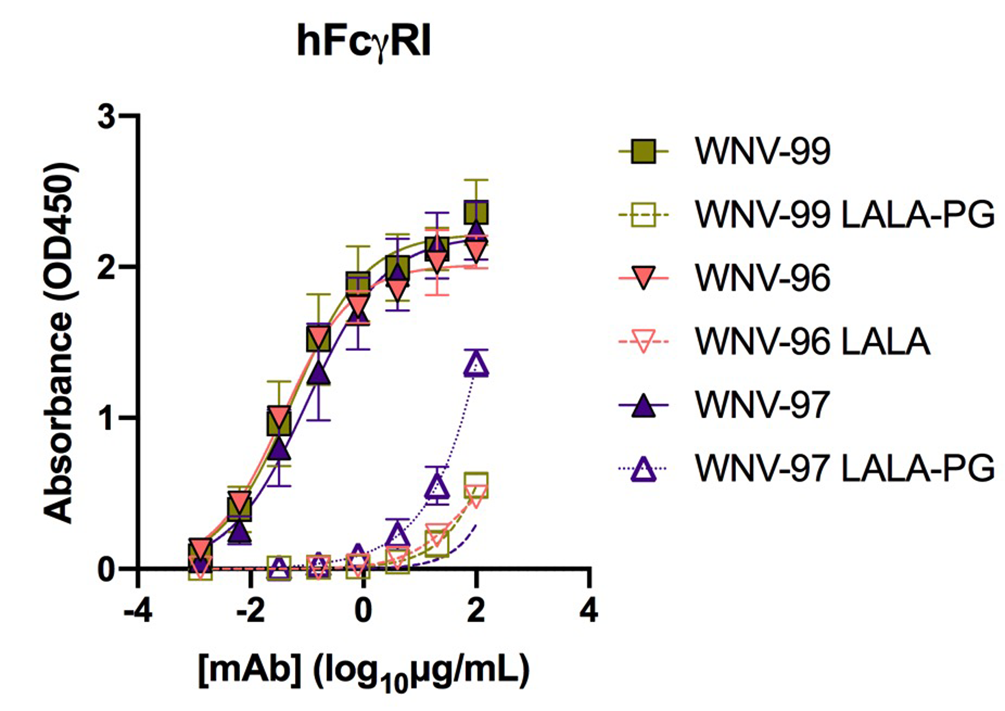

Supplement: FIG S4 [file mbio.02440-21-sf004.tif]
